# The helical domain of the EcoR124I motor subunit participates in ATPase activity and dsDNA translocation

**DOI:** 10.7717/peerj.2887

**Published:** 2017-01-18

**Authors:** Vitali Bialevich, Dhiraj Sinha, Katsiaryna Shamayeva, Alena Guzanova, David Řeha, Eva Csefalvay, Jannette Carey, Marie Weiserova, Rüdiger H. Ettrich

**Affiliations:** 1 Center for Nanobiology and Structural Biology, Institute of Microbiology of the Academy of Sciences of the Czech Republic, Nove Hrady, Czech Republic; 2 Faculty of Sciences, University of South Bohemia in Ceske Budejovice, Nove Hrady, Czech Republic; 3 Institute of Microbiology, Academy of Sciences of the Czech Republic, Prague, Czech Republic; 4 Chemistry Department, Princeton University, Princeton, NJ, United States; 5 College of Medical Sciences, Nova Southeastern University, Fort Lauderdale, FL, United States

**Keywords:** *E. coli*, Multisubunit enzyme complex, Molecular modeling, Domain interactions, DNA restriction enzymes

## Abstract

Type I restriction-modification enzymes are multisubunit, multifunctional molecular machines that recognize specific DNA target sequences, and their multisubunit organization underlies their multifunctionality. EcoR124I is the archetype of Type I restriction-modification family IC and is composed of three subunit types: HsdS, HsdM, and HsdR. DNA cleavage and ATP-dependent DNA translocation activities are housed in the distinct domains of the endonuclease/motor subunit HsdR. Because the multiple functions are integrated in this large subunit of 1,038 residues, a large number of interdomain contacts might be expected. The crystal structure of EcoR124I HsdR reveals a surprisingly sparse number of contacts between helicase domain 2 and the C-terminal helical domain that is thought to be involved in assembly with HsdM. Only two potential hydrogen-bonding contacts are found in a very small contact region. In the present work, the relevance of these two potential hydrogen-bonding interactions for the multiple activities of EcoR124I is evaluated by analysing mutant enzymes using *in vivo* and *in vitro* experiments. Molecular dynamics simulations are employed to provide structural interpretation of the functional data. The results indicate that the helical C-terminal domain is involved in the DNA translocation, cleavage, and ATPase activities of HsdR, and a role in controlling those activities is suggested.

## Introduction

Type I restriction-modification enzymes (RMs) are multisubunit, multifunctional molecular machines that recognize specific, typically asymmetric, DNA target sequences of ∼13–14 bp (Murray, 2002). In sharp contrast to the straightforward mechanisms of the Type II RMs (Loenen et al., 2014), the multisubunit organization of Type I RM systems underlies their multifunctionality. Depending on the methylation status of adenine residues in the target sequence, three Type I subunits either act together as a typical methyltransferase, or recruit a pair of endonuclease motor subunits that initiate translocation of DNA through the enzyme and eventually cleave non-specifically at apparently random sites (Janscak et al., 1999). The protein complex remains bound at the target sequence while up to thousands of bp are pumped through the enzyme by tracking along the duplex helical pitch at rates of up to hundreds of bp per second (Bickle, Brack & Yuan, 1978; Studier & Bandyopadhyay, 1988; Szczelkun et al., 1996; Ellis et al., 1999; Seidel et al., 2004). Translocation is driven by RecA-helicase-like motor subunits that consume ∼1 ATP per ∼1 bp (Seidel et al., 2008) without separating the duplex strands (Stanley et al., 2006). The endonuclease activity of the motor subunits is unmasked under conditions that suggest a role for DNA topology in control of cleavage activity (Yuan, 1981; Janscak et al., 1999; Janscak & Bickle, 2000).

The X-ray crystal structure of the first Type I R-M motor subunit was solved in 2009 for EcoR124I from the *E. coli* plasmid pTrcR124 (Lapkouski et al., 2009). EcoR124I is the archetype of Type I R-M family IC and is composed of three distinct subunit types, HsdS, HsdM, and HsdR, which are encoded by the *hsd* (host specificity of DNA) genes. The fully assembled enzyme complex is a pentamer with R2M2S1 stoichiometry (Janscak, Dryden & Firman, 1998; Mernagh et al., 1998). Small-angle neutron scattering experiments revealed the locations of HsdS and HsdM (Taylor et al., 2010), and electron microscopy and neutron and small-angle X-ray scattering (Kennaway et al., 2012) indicate that DNA binding triggers compaction of the R2M2S1 enzyme from a conformation that has been described as more relaxed, loose, or open. The motor subunit HsdR is composed of four distinct structural and functional domains: an endonuclease domain at the N-terminus with characteristic motifs of the RecB nuclease superfamily (Obarska-Kosinska et al., 2008; Sisáková et al., 2008a; Sisáková et al., 2008b); a C-terminal *α*-helical domain proposed to contact methyltransferase (Davies et al., 1999); and two RecA-like helicase domains situated between the endonuclease and C-terminal domains (Obarska-Kosinska et al., 2008; Lapkouski et al., 2009). Like other members of the SF2 translocase family, EcoR124I translocates on dsDNA (McClelland & Szczelkun, 2004; Firman & Szczelkun, 2000; Seidel et al., 2004) and lacks strand separation activity (Stanley et al., 2006). It has been proposed that helicase domain 1 contacts the 3^′^–5^′^ dsDNA strand tightly during translocation, while helicase domain 2 is proposed to be responsible for DNA advancement through large and rapid conformational changes (Lewis et al., 2008).

**Figure 1:**
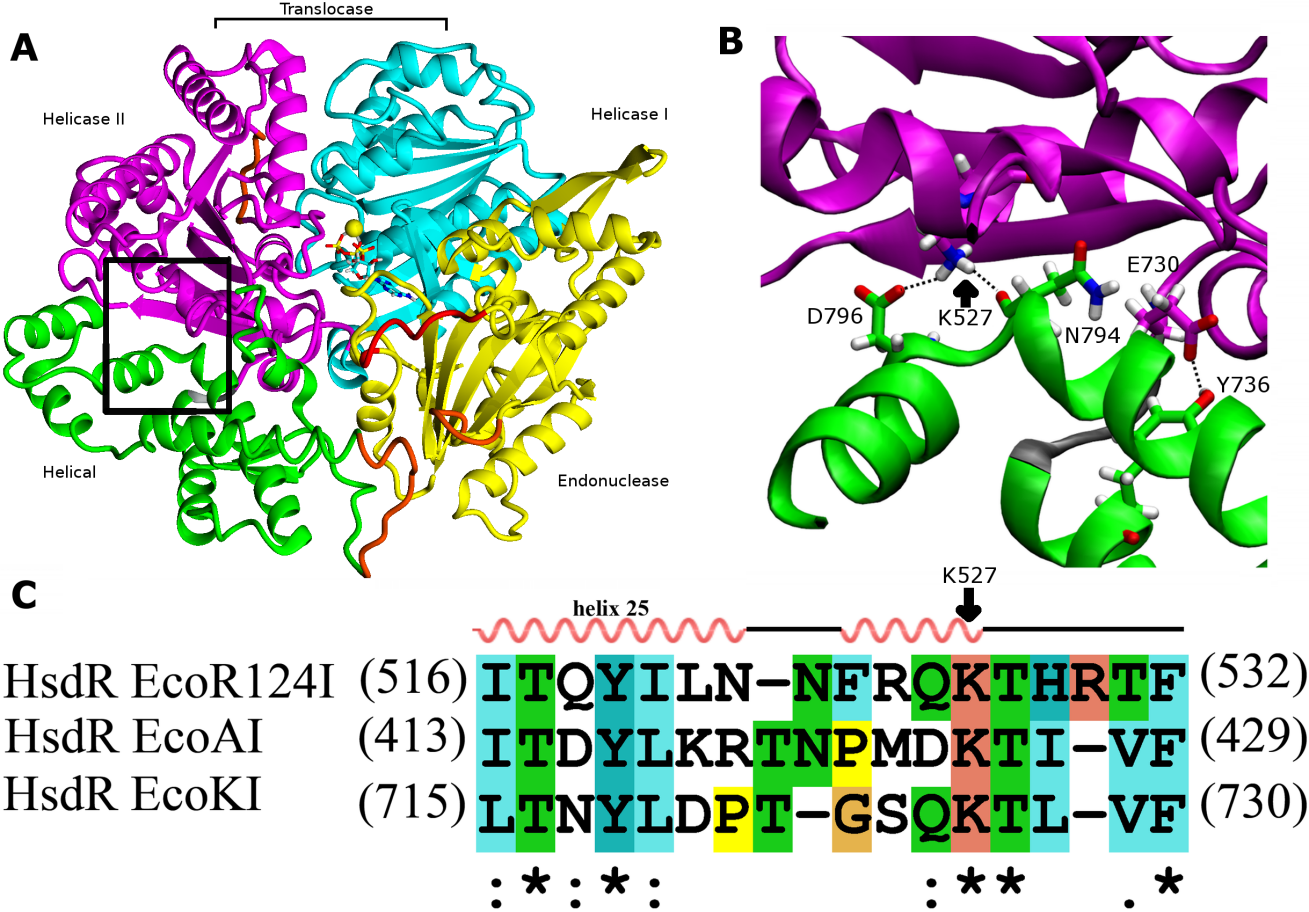
Interdomain interactions at the helical-helicase 2 domain interface in the HsdR subunit of EcoR124I restriction-modification complex. (A) The structure of HsdR consisting of four domains: the endonuclease domain is in yellow, the helicase 1 and the helicase 2 domains are depicted in cyan and magenta, respectively. The C-terminal helical domain is shown in green. ATP is represented in elemental colors as a skeletal model with cyan carbons, and magnesium is indicated by a yellow sphere. The modelled parts of the structure (not resolved in the crystal structure) are in orange and the modeled 180s loop comprising residues 182–189 that is close to the catalytic site on the endonuclease domain is in red just below ATP (coordinates borrowed from K220A mutant HsdR crystal structure ((PDB id: 4BEC, Csefalvay et al., 2015)). The short linker between helicase 2 domain and the C-terminal helical domain is in grey at the lower right corner of the black rectangle (B). Enlargement of the region enclosed by the black rectangle in (A), showing residues involved in interactions at the interface between the helical domain and helicase domain 2. Side chains are shown as skeletal models in atomic colors with carbon atoms colored according to their subunit shown in (A). Hydrogen bonding interactions discussed in the text are indicated with black dotted lines. (C) Alignment of the interface region sequence of EcoR124I from family IC of with sequences from HsdR of the archetypical members of the other two type I R-M families, EcoKI from family IA and EcoAI from family IB. Secondary structures of Eco124I HsdR from the crystal strutcure (pdb code: 2w00) are shown on top. Colors and symbols below the sequences indicate residue similarity: ‘*’ fully conserved residues; ‘:’ highly similar residues; and ‘.’ somewhat similar residues.

Because both the translocase and endonuclease functions are part of the array of integrated structural domains in the large HsdR subunit of 1038 residues, a large number of interdomain contacts might be expected. However, the crystal structure of EcoR124I HsdR (Lapkouski et al., 2009) reveals a surprisingly sparse number of contacts particularly between the helical domain and helicase domain 2. The contact interface between the two domains is only 859 Å^2^. Only a tiny fraction of it, 15 Å^2^, is hydrophobic, and only two potential hydrogen-bonding contacts are found. One is centrally located between the two domains, a bifurcated contact in which the K527 sidechain atom N*ζ* is at a distance of 2.9 Å from the D796 carboxylate O*δ* atom and 2.7 Å from the backbone carbonyl oxygen atom of N794. A second hydrogen bond is possible close to the short loop (K731-T734) that links the two domains, where the phenol O of Y736 is at a distance of 2.6 Å from the Oε2 atom of the E730 carboxylate (Fig. 1). These two hydrogen bonds constitute the only contacts detected at the domain interface. This finding led to a search for sequence similarities in the HsdR subunits of other Type I R-M enzymes that might support the significance of these contacts. The sequence alignment in Fig. 1C suggests that K527 may be part of a new motif that can be defined as (Q/D)KT that is also identified in the HsdR subunits of the EcoKI and EcoAI enzymes that typify Type I R-M families IA and IB. This proposal represents the first extension to all three Type I R-M families of a sequence alignment beyond the conserved DEAD-box motifs of the helicase active sites.

As helicase domain 2 is proposed to be the key player in DNA advancement, undergoing large conformational changes during the translocation cycle (Dürr et al., 2005), the question arises whether these two contacts to the helical domain have a role in the translocation cycle. If so, the helical domain itself could have a previously undescribed role in the translocation cycle beyond its presumed role in interacting with methyltransferase. In the present work, the relevance of these two potential hydrogen-bonding interactions for the translocation activity of EcoR124I is evaluated by analysing mutant enzymes using *in vivo* and *in vitro* experiments. Molecular dynamics simulations are employed to provide structural interpretation of the functional data. The results suggest a role for the helical domain in DNA translocation.

## Materials and Methods

### Mutagenesis, expression and protein purification

Plasmid pTrcR124 (Janscak, Abadjieva & Firman, 1996) carrying the hsdR gene  was used for site-directed mutagenesis. Oligonucleotide primers were used in the  polymerase chain reaction (forward primer sequence is given, reverse primer is its complement; mutated codon underlined, changed nucleotides in bold): 5^′^-TTTCCGCCAG **GC** AACCCACCGTACC-3^′^ for K527A, 5^′^-GAGTTATACAGAG**GC** TATGGAAGGC-3^′^ for Y736A, and 5^′^-GCAAAACTATG **C** TGAATTTGCCACG-3^′^ for D796A. The double mutant K527A_Y736A was obtained by introducing a second point mutation into the mutant plasmid Y736A.

The polymerase chain reaction was performed in 200 µl PCR tubes in an Eppendorf (Hamburg, Germany) mastercycler. The 20-µl reaction mixture contained 1.5 U Expand Long Range polymerase (Roche, Basel, Switzerland); 1X Expand Long Range Buffer with MgCl2; 100 ng of plasmid DNA pTrcR124; 0.3 µM each of the forward and reverse primers; 200 µM dNTPs; and 3 (v/v)% or 6 (v/v)% DMSO. PCR cycles were: 2 min at 96°C;30×(30s at 96°C, 90s at 55°C, 8 min at 68°C); 7 min at 68°C. DpnI (20 U) was used to degrade methylated parental plasmid. The reaction mixture was transformed into *E. coli* DH5*α* competent cells grown on LB-agar plates containing 100 µg/ml of ampicillin. Plasmid DNA was isolated using a Zyppy plasmid miniprep kit (Zymo Research, Irvine, CA, USA) and  sequenced.

*E. coli* BL21(DE3) Gold competent cells were transformed with plasmid DNA containing the desired mutation. Overnight cultures were diluted 1:100 into 0.5 l of fresh LB medium in a 3-l flask supplemented to a final concentration of 100 µg ml^−1^ with ampicillin and incubated at 180 rpm at 37°C until OD600 was about 0.5–0.6. IPTG was added to a final concentration of 1 mM and the culture was incubated for an additional 4 h at 37°C and 30°C for WT and mutant HsdR subunits, respectively. Harvested cells were washed in sodium chloride–Tris-EDTA buffer, pH 8.0. WT and mutant HsdR subunits were purified as described previously (Janscak, Abadjieva & Firman, 1996). EcoR124I methyltransferase was expressed from pAC15M (Holubová et al., 2004) and pJS491 (Patel et al., 1992) plasmids encoding HsdM and HsdS subunits, respectively, and purified as described previously (Taylor et al., 1992). Purified methyltransferase and HsdR subunits were mixed together in a 1:6 ratio to reconstitute the EcoR124I enzyme complex *in vitro*.

### *In vivo* restriction activity assay

*E.coli* strain JM109(DE3) (Yanisch-Perron, Vieira & Messing, 1985) served for complementation analysis of restriction function *in vivo*. For negative complementation the pTrcR124 plasmid expressing WT or mutants HsdR was transformed into *E. coli* JM109(DE3)[pKF650], a restricting (r^+^m^+^) host containing all three *hsd* genes of EcoR124II. For positive complementation pTrcR124 was transformed into *E.coli* JM109(DE3)[pACMS] expressing only the EcoR124II methyltransferase (r^−^m^+^) (Patel et al., 1992). Soft agar at 45°C was mixed with 0.5 ml of each fresh overnight culture, gently mixed, and immediately poured onto agar plates with appropriate antibiotics. The virulent mutant of phage *λ* was used for testing of restriction phenotype (Jacob & Wollman, 1954). Solidified soft agar was spotted with 30 µl each of tenfold serial dilutions of *λ*vir.0 phage lysate at 10^2^ to 10^6^ plaque-forming units/ml. The spots were dried at room temperature and the plates incubated overnight at 37°C (Colson et al., 1965). Phage buffer, complex LB medium and *in vivo* restriction assays were as described (Hubácek, Holubová & Weiserová, 1998; Sisáková et al., 2008b). The solid medium is LB with agar added at 1.5 (w/v)%. Soft agar overlay is LB with agar added at 0.6 (w/v)%. Antibiotics were used at the following concentrations: ampicillin; 100 mg ml^−1^, chloramphenicol; 50 mg ml^−1^. The efficiency of plating was determined as the number of plaques on the tested strains compared to the number of plaques on the non-restricting control strain *E. coli* JM109(DE3) (Yanisch-Perron, Vieira & Messing, 1985). Values in the range 0.0001–0.01 correspond to the restriction-competent (r^+^) phenotype, those in the range 0.01–0.1 to the mixed-competence (r±) phenotype, and those in the range 0.1–1 to the restriction-deficient (r^−^) phenotype.

### *In vitro* cleavage activity assay

Cleavage activity was assayed *in vitro* using supercoiled plasmid DNA substrate pRK (Taylor et al., 1992) carrying a single recognition site for EcoR124I. Cleavage activity was assayed (Janscak, Abadjieva & Firman, 1996) at 37°C in restriction buffer (50 mM Tris–HCl, pH 8.0, 1 mM DTT, 10 mM MgCl2 and 50 mM NaCl) containing 15 nM of the circular pRK plasmid DNA, 15 nM methyltransferase and 90 nM HsdR. After 1 min of pre-incubation the reaction was started by addition of ATP and SAM to a final concentration 4 mM and 0.2 mM, respectively. The reaction was stopped by adding 0.25 vol of stop reagent (3 (w/v)% SDS; 0.15 M EDTA; 10 (w/v)% glycerol; 0.1 (w/v)% bromophenol blue) and heating at 65°C for 5 min. Samples were loaded into 1 (w/v)% agarose gels in Tris-acetate-EDTA buffer and run at 5 V/cm for 130 min. Gels were stained with 2 µg/ml ethidium bromide, destained in water and photographed under UV illumination. The relative amounts of DNA were evaluated by densitometry using ImageJ software (Abramoff, Magalhaes & Ram, 2004).

### DNA Translocation assay—steady state analysis

The method utilizes a fluorescently labeled triplex forming oligonucleotide (TFO) attached at a specific distance from the EcoR124I binding site on plasmid DNA. Translocation by EcoR124I leads to the dissociation of the triplex, which can be monitored by the change in fluorescence upon displacement (Firman & Szczelkun, 2000; McClelland, Dryden & Szczelkun, 2005). Plasmid pLKS5 carrying a DNA triplex binding site 1,517 bp downstream of the EcoR124I recognition site was used (Stanley & Szczelkun, 2006). Linear DNA for the triple-helix displacement assay was generated by ApaI digestion of pLKS5 plasmid DNA followed by phenol/chloroform extraction, chloroform extraction and isopropanol precipitation. The triplex was formed by incubating 50 nM linear and 25 nM 5^′^-end Tetramethylrhodamine (TAMRA) labeled oligonucleotide 5^′^-‘TTTCTTCTTCTTTTCTTTTCTT-3’ (Eurofins MWG Operon) in buffer (10 mM MES pH 5.5, 12.5 mM MgCl_2_) at 23°C overnight. Following overnight incubation, the triplex was stored on ice, and then diluted 1/10 in reaction buffer (50 mM Tris–HCl, pH 8.0, 1 mM DTT, 10 mM MgCl2 and 50 mM NaCl) before use. 5 nM Triplex was pre-incubated with 40 nM MTase and 120 nM wt or mutant HsdR at RT °C for 5 min. Following Simons & Szczelkun (2011) the required concentration of the HsdR subunit for R_2_M_2_S_1_ complex formation is twice that of the DNA under the given conditions. A 120 nM concentration of HsdR (methyltransferase (M_2_S_1_) to HsdR ratio 1:3) was enough to saturate both methyltransferase binding sites under experimental conditions. Translocation reaction was initiated with 4 mM ATP and after 10 min triplex displacement was measured. Steady state anisotropy for the bound (triplex) and free forms of TAMRA labeled TFO was measured using a Tecan Safire 2 Multi-detection Microplate Reader. Excitation and emission wavelengths used were: 544 and 576 nm, bandwidths were kept <10 nm. Data are expressed as the means of three independent experiments.

### ATPase activity assay *in vitro*

ATPase activity was assayed using a radioactivity-based ATPase assay. In a first step cellulose plates were prepared by marking positions for sample drops with a pencil: 1.5 cm from the bottom of the plate and 1 cm in between dots. A total of 50 ml of running buffer (0.4 M LiCl, 1 M Formic acid) or water was carefully poured in the glass chamber, avoiding drops on the walls. At first each cellulose plate was pre-run in running buffer and dried. Then, the same steps were performed with destilled water. Prepared plates can be stored at room temperature up to one week.

ATPase activity itself was assayed in NEB2 buffer containing 10 mM Tris–HCl, pH 7.9, 10 mM MgCl_2_, 50 mM NaCl, 1 mM DTT; 15 nM methyltransferase, 90 nM HsdR, and 6 molar excess (90 nM) of covalently closed circular DNA pRK containing single recognition site for EcoR124I to prevent DNA cleavage. Excess of DNA concentration over enzyme was used (6:1 ratio) following Janscak, Abadjieva & Firman (1996) and 6-folds excess in HsdR subunit concentration ensures efficient ATPase activity as was described by Janscak, Dryden & Firman (1998). The HsdR subunit does not turn over after cleavage of supercoiled DNA that might occur, nevertheless, ATPase activity is efficient under tested conditions (Simons & Szczelkun, 2011). Reactions (40 µL) were started by addition of ATP mixture containing 0.16 µCi (0.0013 mM) [*γ*^32^P]-ATP to a final concentration of 2 mM and incubated at 37°C. Aliquots (4 µL) were taken at the indicated time points and stopped by adding 1 (w/v)% SDS. The hydrolyzed ^32^P_i_ was separated from [*γ*^32^P]-ATP by cellulose TLC in 0.4 M LiCl_2_, 1M formic acid (Randerath & Randerath, 1964) and the distribution of radioactivity between ^32^P_i_ and ATP was visualized using a Fujitsu 9000 scanner (Marini & Krejci, 2012).

### Molecular dynamics simulations

The WT crystal structure of the motor subunit HsdR from the restriction-modification system EcoR124I (PDB entry 2W00) and a recent crystal structure of HsdR mutant Lys220Ala (PDB entry 4BEC) were used for preparing the structural model for all simulations. Three missing segments from the WT crystal structure (residues 142–147, 585–590, and 859–869) were built using standard loop modeling in YASARA (Krieger, Koraimann & Vriend, 2002; Konagurthu et al., 2006) and added to the WT crystal structure. The missing segment from residue 182 to 189 is resolved in the Lys220Ala mutant crystal structure and thus was built into the WT structure by adding the coordinates from the mutant to the above modeled structure, followed by steepest-descent energy minimization. All classical MD simulations were performed using GROMACS 4.64 (Berendsen, Van der Spoel & Van Drunen, 1995; Van der Spoel et al., 2005; Pronk et al., 2013) with the AMBER99SB forcefield (Hornak et al., 2006). ATP was parameterized by applying the standard RESP procedure using Antechamber (Wang et al., 2004), where charges for free MgATP were derived from HF/6-31G* calculation in Gaussian03 (Frisch et al., 2004). Histidine was assumed to be charged, with the ND and NE atoms protonated; arginine and lysine residues were assumed to be protonated. Mutants were prepared *in silico* by replacing the respective side-chain in YASARA followed by short minimization of 100 ps with freezing of all residues except those close to each point mutation, to avoid any local crashes in the side-chain and then simulated for 100ns using classical molecular dynamics simulations and analyzed to evaluate possible conformational changes. All systems were solvated in explicit TIP3P water (Jorgensen et al., 1983) in a cubic box with a margin of 10 Å and neutralized by adding sodium counterion. The particle-mesh Ewald method (Darden et al., 1993) was applied to calculate long-range electrostatic interactions with a cutoff distance of 10 Å and a Lennard-Jones 6–12 potential was used to evaluate van der Waals interactions within 10 Å cutoff distance. The LINCS algorithm of fourth order expansion was used to constrain bond lengths (Hess et al., 1997). After solvation and neutralization each system was energy minimized for 10,000 step using steepest descent optimization method to remove poor van der Waals contacts in the initial geometry. After minimization two stages of equilibration were conducted. Firstly the system was equilibrated for 1 ns with position restraints of 10,000 kJ/mol on all heavy atoms. A constant temperature of 300 K was maintained using the V-rescale algorithm (Bussi, Donadio & Parrinello, 2007) with a coupling time of 0.1 ps and separate baths for the solute and the solvent. The pressure was kept constant at 1 bar using the Parrinello-Rahman pressure coupling scheme (Parrinello & Rahman, 1981) with a time constant of 2 ps. Initial velocities were generated randomly using a Maxwell–Boltzmann distribution corresponding to 300 K. Neighbor lists were updated every 10 fs using a group cut-off scheme. Finally the production run was performed for 100 ns without restraints at 300 K in the isothermal–isobaric ensemble.

Principal-component analysis (Amadei, Linnssen & Berendsen, 1993) was used using g_covar and g_anaeig tools in the GROMACS package to identify the global motions of the system using backbone atom only. The g_rms tool of the GROMACS package was used to calculate root mean square deviations (RMSD) during the trajectories taking the minimized crystal structure as reference. Root mean square fluctuations (RMSF) of the backbone of each residue were calculated by g_rmsf while atomic distances were measured by g_dist.

## Results

### Restriction activity *in vivo*

The restriction activity of wild-type (WT) HsdR and all mutants was measured *in vivo* by monitoring the ability of cells expressing WT HsdS-HsdM2 methyltransferase and WT or mutated HsdR to restrict the growth of unmodified bacteriophage *λ*vir.0. Positive and negative complementation tests (Sisáková et al., 2008b), respectively, were used to distinguish between defects in DNA cleavage and defects in the interaction of mutant HsdR subunits with methyltransferase. Positive complementation uses a restriction-deficient (r^−^) host lacking WT HsdR to test if a mutant HsdR subunit functions in DNA cleavage. Negative complementation uses a restriction-competent (r^+^) host expressing WT HsdR to test if a mutant HsdR subunit that is defective in DNA cleavage is competent for assembly with methyltransferase, allowing it to compete with WT HsdR subunits and thus reduce their restriction activity. This effect is called a trans-dominant effect. Wildtype HsdR complements restriction in the r^−^ host in the positive complementation test (Table 1, r^−^ host), reducing *λ*vir.0 infectivity as expected (value 0.001, within the range 0.0001–0.01 expected for the restriction-proficient phenotype REF). All four mutants, K527A, Y736A, K527A_Y736A, and D796A, fail to complement restriction in the r^−^ host (all values within the range 0.1–1 expected for the restriction-deficient phenotype) and are therefore restriction-deficient. In the negative complementation test the reduced restriction activity in the r^+^ host (Table 1, r^+^ host) indicates that all four mutant subunits are fully competent for assembly with methyltransferase to form the endonuclease complex. The *in vivo* results thus support a role for the helical domain in enzyme activity.

**Table 1:** Effect of mutations on the restriction phenotype of EcoR124I.

HsdR	Restriction^a^		Ability of cleavage	Ability of assembly
	r^−^ host^b^	r^+^ host^c^		
WT	0.0012 ± 0.0005^SD^	0.0011 ± 0.0004	Yes	Yes
K527A	0.4388 ± 0.3009	0.1092 ± 0.0857	No	Yes
Y736A	0.3197 ± 0.2147	0.2307 ± 0.0694	No	Yes
K527A + Y736A	0.4230 ± 0.1953	0.1619 ± 0.0666	No	Yes
D796A	0.3962 ± 0.0885	0.1965 ± 0.1335	No	Yes

**Notes.**

a Restriction activity was determined as the efficiency of plating of *λ*vir.0 on tested strains relative to the efficiency of plating of *λ*vir.0 on *E. coli* JM109(DE3) indicator (nonrestricting) strain.

b The positive complementation was tested in r^−^ host *E. coli* JM109(DE3)[pACMS] (r^−^m^+^).

c Negative complementation (transdominant effect) in r^+^ host *E. coli* JM109(DE3)[ pKF650] (r^+^m^+^).

SD, standard deviation.

### DNA cleavage activity *in vitro*

DNA cleavage activity is tested *in vitro* on a covalently closed circular plasmid DNA containing a single EcoR124I recognition site using the EcoR124I RM complex reconstituted with either WT or mutant HsdR. DNA cleavage *in vitro* proceeds in two steps (Janscak, Abadjieva & Firman, 1996). When translocation is impeded the enzyme first cleaves one strand of the DNA duplex resulting in a nicked DNA product, which is then linearized in a second step. Although cleavage is non-specific and distant from the single recognition sequence, the resulting linearized DNAs are all of the same length. For efficient DNA cleavage a 1:1 ratio of reconstituted EcoR124I R2M2S1 complex to DNA and 6:1 ratio of HsdR subunits to methyltransferase (M_2_S_1_-complex) was used. An excess of HsdR subunit over methyltransferase is required for successful formation of restriction-proficient complexes with stoichiometry of R_2_M_2_S_1_ because the second HsdR subunit binds to methyltransferase with at least two orders of magnitude lower affinity than the first one (Janscak, Dryden & Firman, 1998; Mernagh et al., 1998).

**Figure 2:**
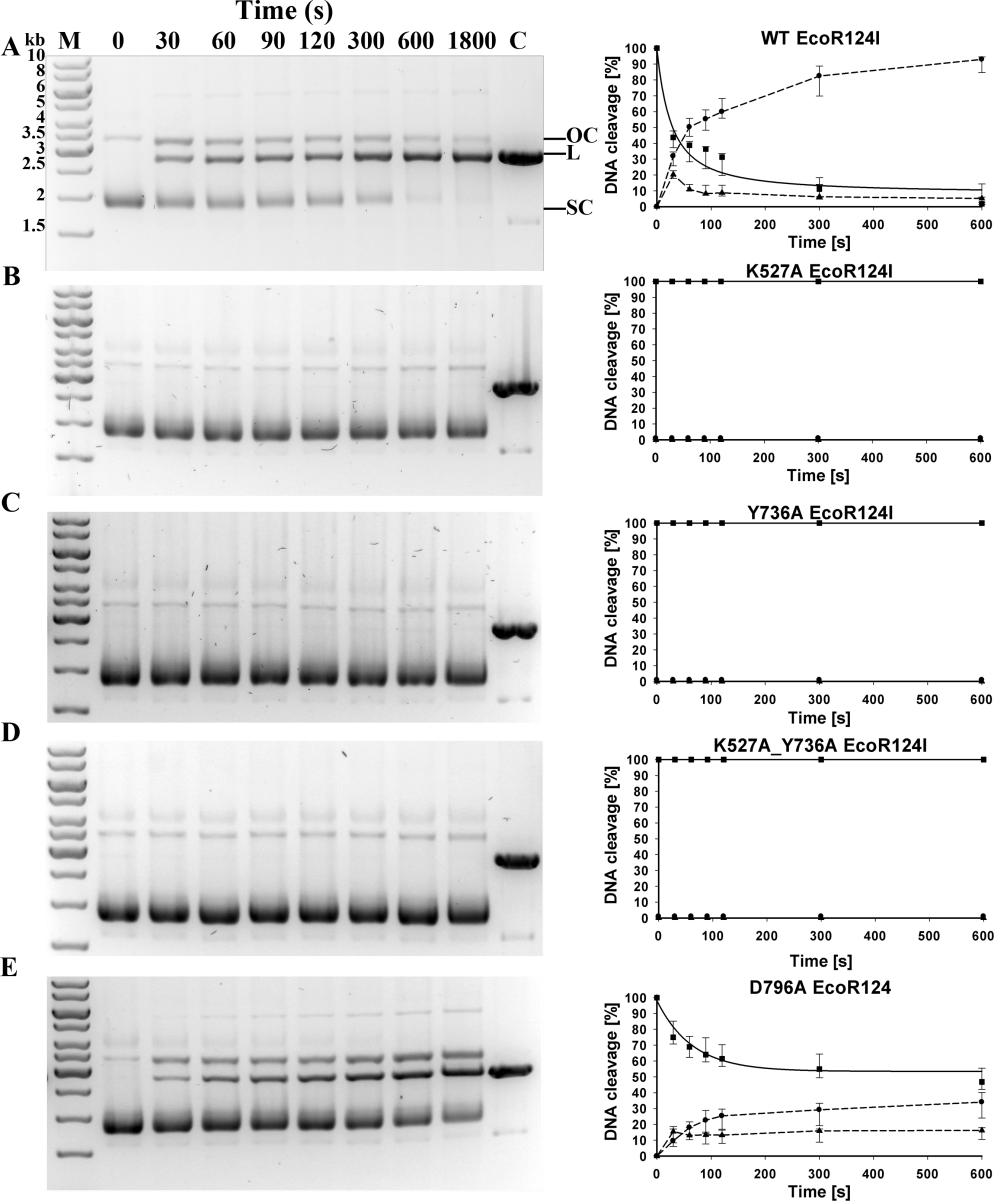
Cleavage of supercoiled substrate. (A) Supercoiled DNA substrate plasmid pRK carrying single EcoR124I recognition site is cleaved by EcoR124I R-M complex *in vitro*. EcoR124I was reconstituted from HsdS1HsdM2 methyltransferase and (A) WT HsdR or mutant HsdRs (B) K527A, (C) Y736A, (D) K527A_Y736A and (E) D796A. Aliquots were quenched at the time points indicated in seconds and resolved on agarose gels stained with ethidium bromide. On (A) open circle and linear products are denoted as OC and L, respectively, and supercoiled substrate as SC; control (plasmid pRK linearized by HindIII restriction enzyme) is denoted as C; DNA molecular weight marker (M) with marked band size in kb on (A). (B) Quantification. DNA species were quantified using ImageJ software (Abramoff, Magalhaes & Ram, 2004). Gels were scanned under UV illumination, and the image converted to grey scale and then inverted. The band density in the lane at time zero (before initiation of the reaction with ATP) is taken as 100% of supercoiled substrate. The three indicated DNA species OC, open circular product (▴); L, linear product (●); SC, supercoiled substrate (■) were quantified individually from densities of bands in the gels on the left panels. Error bars represent standard deviations calculated from at least three independent experiments for each enzyme. Rates for the decrease of supercoiled DNA substrate were derived by fitting an exponential decay function to the data in SigmaPlot (solid lines); dashed lines connect the points only to guide the eye and do not represent fits to the data.

With reconstituted WT enzyme in 30 min of incubation, essentially all the circular substrate with a single EcoR124I recognition site is cleaved on one or both strands, generating approximately 90% linear product and 10% nicked DNA (“open circle” on Fig.  2). Mutants K527A, Y736A, and the double mutant K527A_Y736A are unable to cleave this circular DNA. Mutant D796A exhibits measurable but significantly decreased cleavage activity compared to WT, with ∼47 % of the DNA remaining circular, ∼35 % cleaved completely, and ∼18 % nicked. To quantify the relative cleavage rates of different mutant proteins, rate constants for the disappearance of supercoiled DNA were estimated for WT and mutant D796A by fitting the data on Fig. 2 with an exponential function. The best-fit value of the rate constant *λ* for the reconstituted WT enzyme is 0.0238 s^−1^, whereas the rate constant *λ* for mutant D796A is approximately two thirds the WT value, 0.0154 s^−1^. The difference between WT and mutant values is in a similar range as found previously for other mutants that display a restriction-deficient phenotype *in vivo* (Csefalvay et al., 2015). Thus the *in vivo* results can be considered a more sensitive indication of enzyme activity than the *in vitro* results.

**Figure 3:**
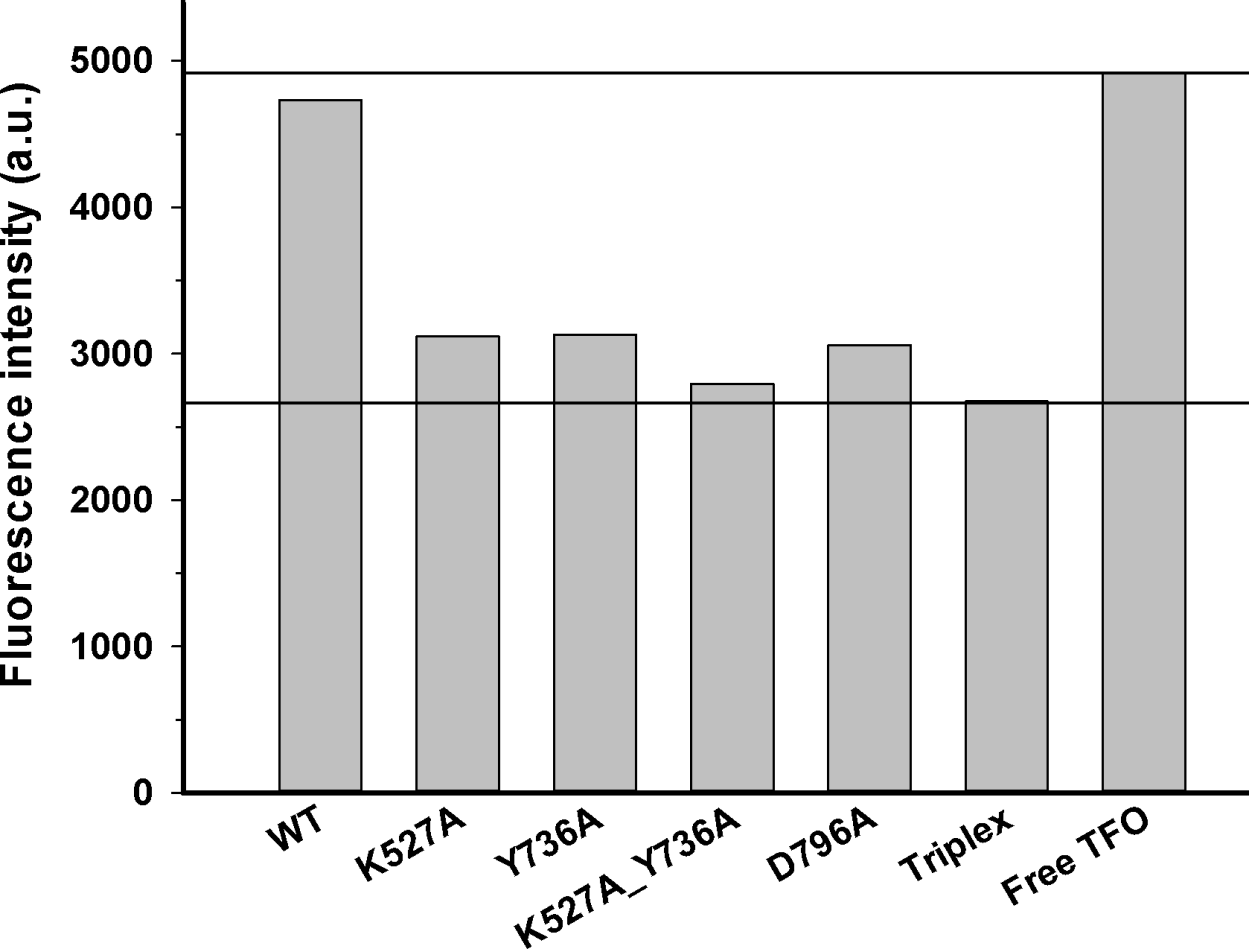
DNA translocation analysis by triplex displacement. Triplex-forming oligonucleotide (TFO) fluorescently labeled with TAMRA is hybridized to *linearized* DNA and translocation is initiated by addition of reconstituted enzyme and ATP as described in Methods. Fluorescence intensities after 10 min reaction time are shown. Bound (triplex) and free (displaced) TFO are indicated by lower and upper horizontal lines, respectively. From left to right, enzyme reconstituted with: wildtype HsdR, mutant K527A HsdR, mutant Y736A HsdR, double mutant K527A_Y736A HsdR, mutant D796A HsdR, unreacted triplex only, and free TFO. No triplex displacement is observed in the absence of ATP or HsdR (not shown). Average values of fluorescence intensities calculated from four or more independent experimental replicates for each individual WT or mutant enzyme species are shown.

### Translocation activity *in vitro*

Translocation of duplex DNA by reconstituted WT and mutant enzymes was measured using a triplex displacement assay. In this assay, a fluorescently-labeled oligonucleotide forms a DNA triplex 1517 bp downstream of the recognition sequence and is displaced when this region of the DNA passes through the enzyme during translocation (McClelland, Dryden & Szczelkun, 2005). Complete displacement of the oligonucleotide by the enzyme results in a fluorescence signal similar to that of the free oligonucleotide. At the DNA to methyltransferase ratio of 8:1 and saturating concentration of HsdR subunits used in this assay, the single recognition site on the circular DNA substrate is expected to be fully bound by the pentameric complex of EcoR124I with stoichiometryR_2_M_2_S_1_ (McClelland, Dryden & Szczelkun, 2005). In control experiments no triplex displacement is observed in the absence of ATP or HsdR. Only the enzyme complex reconstituted with WT HsdR results in fluorescence intensity similar to that of the free oligonucleotide. All four mutant enzymes, K527A, Y736A, D796A, and K527A_Y736A, showed fluorescence intensities similar to that of the initial DNA triplex with bound oligonucleotide (Fig. 3), indicating that these enzymes do not displace the oligonucleotide and thus have lost the ability to translocate duplex DNA. These results demonstrate that these point mutations in the helical domain affect translocation.

### ATP hydrolysis *in vitro*

**Figure 4:**
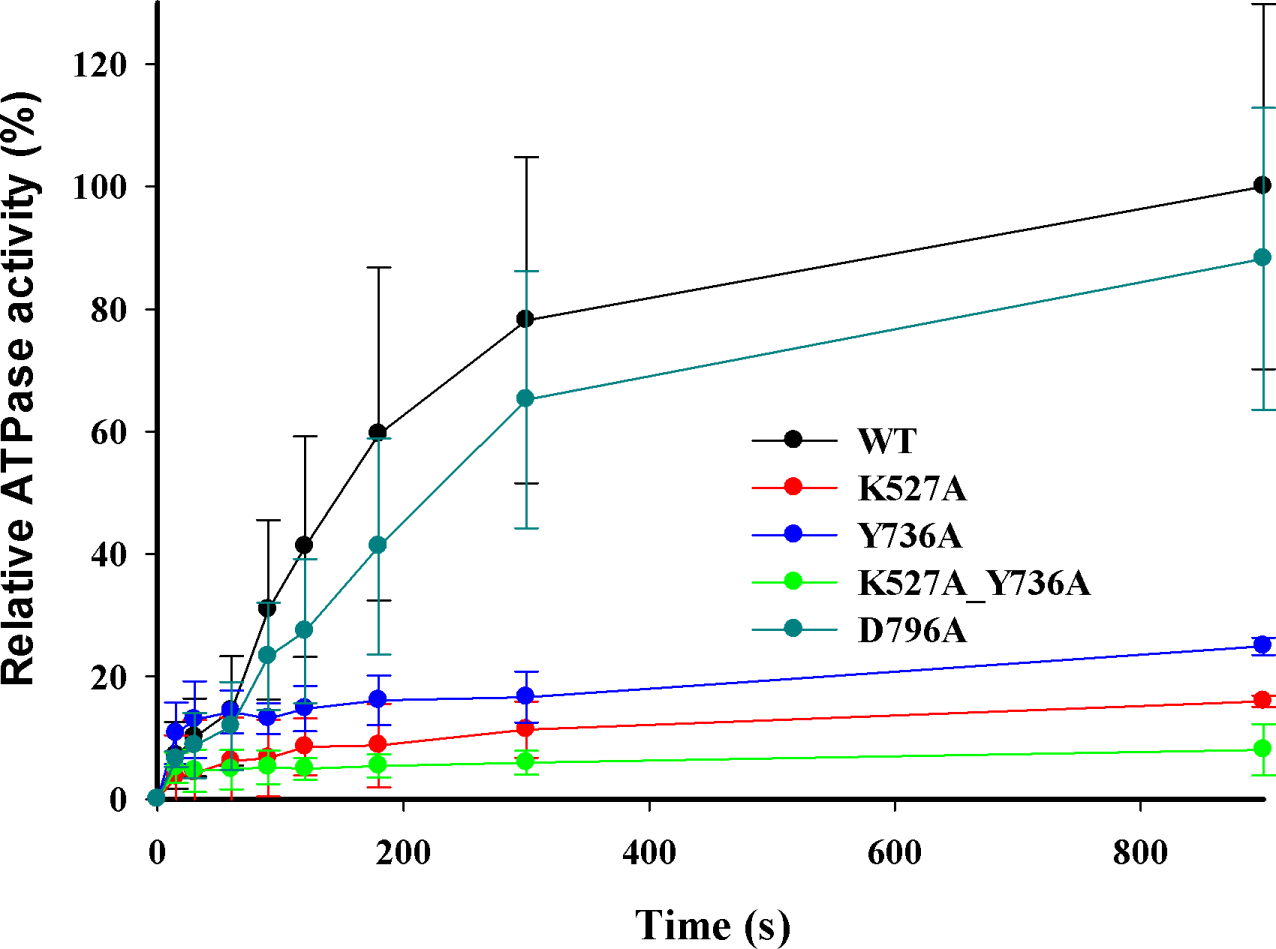
DNA-dependent ATPase activity. EcoR124I reconstituted from methyltransferase and HsdRs WT (black), or mutant K527A (red), Y736A (blue), D796A (green) or K527A_Y736A (dark cyan) was incubated at a final concentration of 15 nM with 90 nM circular plasmid DNA containing one recognition site and 2 mM ATP containing 0.16 µCi g-32P-ATP. At the indicated time points ATP and inorganic phosphate were resolved on cellulose TLC, autoradiographed, and scanned to quantify the extent of hydrolysis. The amount of ATP degraded is plotted as a function of time. Average values and standard deviations are calculated from at least three independent replicates for each mutant enzyme and six for WT enzyme. Data for mutants are normalized to the average value for WT at 900 s taken as 100%.

DNA-dependent ATPase activities were measured using the circular DNA substrate containing a single EcoR124I recognition site after reconstitution of the purified WT and mutant HsdR subunits with HsdS-HsdM_2_ methyltransferase. Control assays omitting either DNA or methyltransferase indicate negligible ATP hydrolysis. A relative ATPase activity of 100% was assigned to the enzyme reconstituted with WT HsdR. The relative ATPase activity of reconstituted mutant enzymes Y736A, K527A, and double mutant K527A_Y736A is ∼25%, 16%, and 8%, respectively (Fig. 4), indicating that these enzymes hydrolyze ATP very poorly *in vitro*, consistent with their inability to translocate duplex DNA *in vitro*. However, reconstituted mutant enzyme D796A exhibits a relative ATPase activity of ∼88%, in apparent contradiction to its lack of translocation activity in the triplex-displacement experiments. The finding that mutant D796A retains ATPase activity but lacks translocation activity suggests that the correlation between these two activities may be weaker than has been previously assumed (Abadjieva & Firman, 1996). Although ATPase activity has been considered an indicator of translocation activity and is a necessary condition for translocation, ATPase activity is only one component of translocation activity, and in this case it is not sufficient for translocation. The decoupling of ATPase activity from translocation in the D796A mutant may reflect structural or dynamic changes that cannot be further described at present. Alternatively or additionally, the different temperatures for the translocation and ATPase assays might be related to the inability to translocate efficiently despite ATPase activity. Under the conditions tested here, although the D796A mutant was able to partially cleave the supercoiled DNA substrate *in vitro*, the rate of DNA cleavage was insufficient for cells to survive phage infection *in vivo*. Nevertheless, the inefficient ATP hydrolysis by three of the enzymes reconstituted with mutant HsdR subunits indicates an unexpected role for the helical domain in ATP hydrolysis.

### Molecular dynamics simulations

The initial structure of the HsdR subunit used in the simulations presented here (Sinha et al., 2014) is based on the published crystal structure (PDB id: 2W00, Lapkouski et al., 2009) with modeled missing loops (residues 142–147, 585–590, and 859–869) built using standard loop modeling in YASARA (Krieger, Koraimann & Vriend, 2002; Konagurthu et al., 2006). This structure has been shown previously to be stable in MD simulations, and the resulting trajectories achieve equilibrium after 50 ns of simulation (Sinha et al., 2014) allowing simulations of 100 ns, which is sufficient to test the effect of point mutations. Structures of single mutants K527A, D796A, and Y736A, and double mutant K527A_Y736A were prepared by swapping each individual residue for the WT residue of the starting structure in YASARA. The trajectories of WT and mutant HsdR subunits reveal that in all cases the secondary structure is maintained throughout and the fold is preserved. In all simulations the root mean square deviations (RMSD) from the starting structure reach a plateau of ∼3 to 3.5 Å after 50 ns, comparable to equilibration times and RMSD values reported previously for the WT HsdR system with modeled missing loops (Sinha et al., 2014) and acceptable for a system of this size having multiple domains.

**Figure 5:**
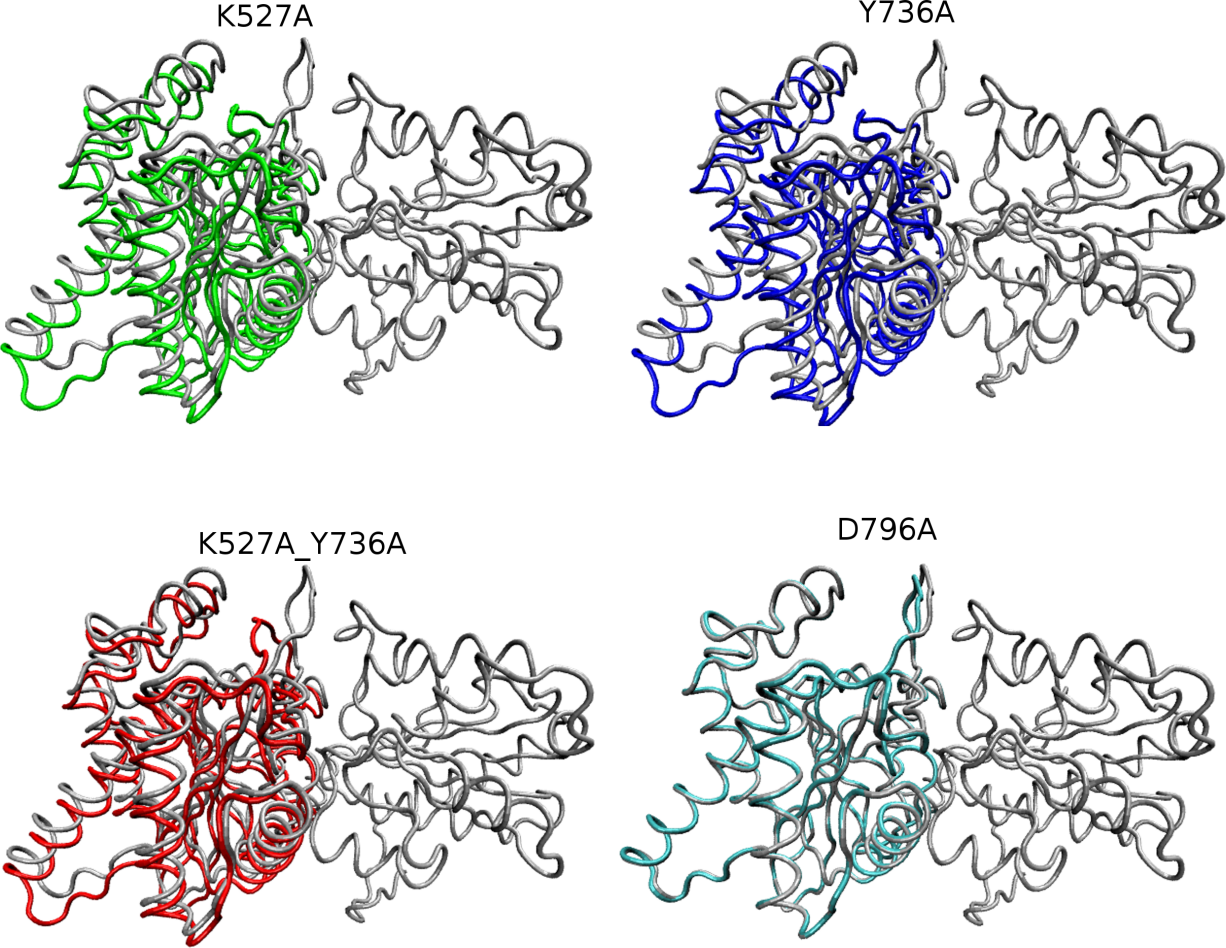
Overlay of WT and mutant structures. Helicase domains 1 and 2 are shown in tube representation with the WT conformation in gray. Mutant conformations are overlaid on helicase domain 1, which is thus identical in all cases and therefore shown only once for better visualization. The position of each helicase 2 domain is depicted in color for each mutant (K527A, green; Y736A, blue; K527A_Y736A, red, and D796A, cyan). Mutant D796A domain 2 overlays substantially with WT, whereas the other three mutants are shifted due to the cleft opening discussed in the text.

**Figure 6:**
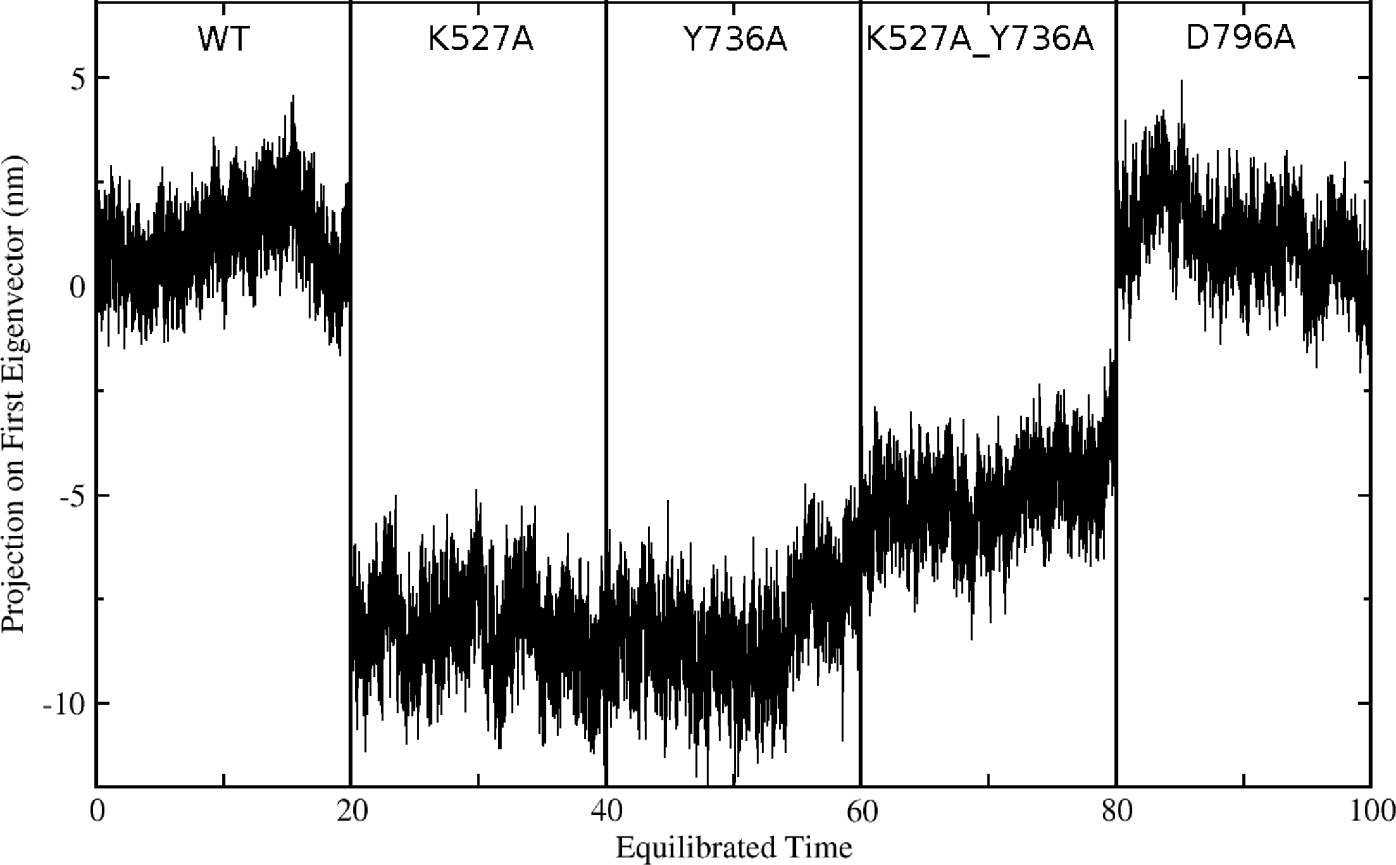
Projection of the first eigenvector of the joined trajectories in the final 20 ns of the equilibrated part of each individual simulation. For analysis the 20 ns segments from the simulation of each protein were joined to make the composite trajectory shown. Data for the WT simulation are represented from 0 to 20 ns, for K527A from 20 to 40 ns, for Y736A from 40–60 ns, for K527A_Y736A from 60 to 80 ns, and for D796A from 80 to 100 ns.

Global motions during the simulations were analyzed by principal components analysis (PCA). Equilibrated trajectories for the final 20 ns of each individual WT or mutant simulation were extracted and joined into one combined trajectory to allow calculation of the first eigenvector that identifies the motion with the largest change (Fig. 5). The global collective motion described by the first eigenvector for mutants K527A, Y736, and K527A_Y736A is a partial opening of the cleft between helicase domains 1 and 2, due mainly to movement of helicase domain 2. Mutant D796A and WT HsdR maintain the starting conformation of the WT subunit, in which the cleft remains closed. Projections along the first eigenvector were analyzed to quantify these structural changes (Fig. 6). These projections describe the extent to which the motion described by the first eigenvector is present in the simulation of each protein. WT HsdR shows no major change along the first eigenvector and its projection value oscillates around zero, as expected because the initial WT structure represents an energy-minimized and equilibrated structure. Like WT HsdR, mutant D796A shows no change along the first eigenvector and its projection oscillates around zero, indicating no opening of the cleft between the two helicase subunits. In contrast, K527A, Y736A, and double mutant K527A_Y736A move ∼10 +∕ − 2 Å along the first eigenvector, indicating considerable movement of helicase domain 2 away from helicase domain 1 to open the cleft compared with the initial crystal structure.

These global structural changes of helicase domain 2 during the simulations are also reflected in the rmsd values calculated for C-alpha atoms by superimposing the last snapshot of each simulated structure on the WT crystal structure. During 100ns of WT MD simulation helicase domain 2 stays in a position almost identical to that in the crystal, with a maximum rmsd of ∼0.2 Å. Significant structural changes occur during the simulations of mutants Y736A, K527A, and double mutant K527A_Y736A, with maximum rmsd values of ∼4.1 Å, ∼3.8 Å and ∼2.8 Å, respectively. Mutant D796A shows a maximum rmsd of ∼0.6 Å, indicating only a minor conformational change similar to that observed in WT. The interdomain distances between centers of mass of the two helicase domains confirm the helicase cleft opening in mutants K527A, Y736A, and K527A_Y736A, whereas in mutant D796A the two domains are somewhat closer to each other than in WT (Table  2).

These results suggest that loss of the limited interdomain contacts between the helical domain and helicase domain 2 leads to substantial structural changes in mutants K527A, Y736A and double mutant K527A_Y736A, characterized by opening of the cleft between the two helicase domains located ∼25 Å away from the site of these mutations. Such a long-range effect is further indication of an integral role for the helical domain in HsdR function. In contrast, in WT and mutant D796A HsdR, helicase domain 2 largely maintains the conformation observed in the WT crystal structure. This result suggests that in the D796A mutant K527 may be able to maintain at least partial contact with the helical domain even though D796 is no longer available as a partner. To further evaluate this possibility a more subtle interaction analysis of the simulation results was carried out.

**Figure 7:**
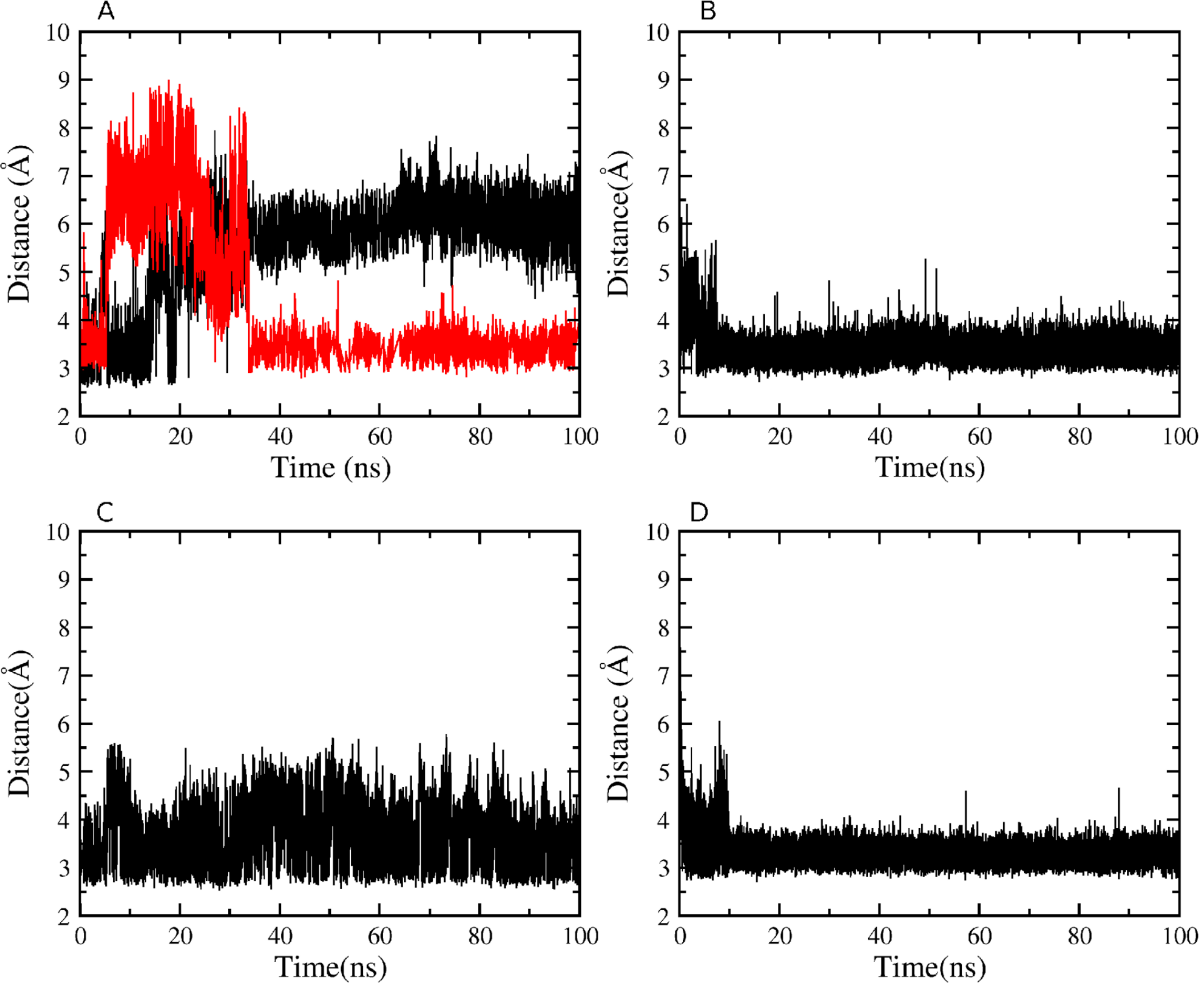
Atomic distances during the 100 ns simulations. (A) WT K527(Nz)-N794(O) in black, WT K527(Nz)-D796(CG) in red; (B) WT Y736(OH)-E730(CD); (C) mutant D796A K527(Nz)-N794(O); (D) mutant D796A Y736(OH)-E730(CD).

The persistence of the interdomain contacts observed in the crystal structure was analyzed in the equilibrated part of each simulation. Although neither H atoms nor hydrogen bonds are detectable in crystal structures, H atoms are included in the calculations based on predicted covalent geometries (Berendsen, Van der Spoel & Van Drunen, 1995). Hydrogen bonds are observed between the K527 amino group and two acceptors, the backbone carbonyl O atom of N794 and the carboxylate O*δ* atom of D796, and between the hydroxyl group of Y736 and carboxylate Oε atom of E730 in the short loop linking the two domains. Figure 7A reveals that during simulation of WT HsdR the hydrogen bond between the K527 amino group and the backbone carbonyl O atom of N794 is lost completely after 20 ns, whereas the hydrogen bond between the amino group of K527 and the carboxylate O*δ* atom of D796 is maintained. The hydrogen bond between the Y736 hydroxyl and the E730 carboxylate is maintained throughout the simulation in WT (Fig. 7B). In mutant D796A the hydrogen bond between the K527 amino group and the D796 carboxylate is no longer possible. The contact between the K527 amino group and the N794 backbone carbonyl O atom is maintained after the initial equilibration time (Fig. 7C), as is the interaction between the Y736 hydroxyl and the E730 carboxylate (Fig. 7D). The resulting conformation of mutant D796A is thus very similar to WT throughout the simulation, showing no large-scale domain movement although its interactions at the helical-helicase interface differ locally.

**Table 2:** Interdomain distances and H-bonding between helicase I and helicase II domains.

	Avg. Inter-domain distance (Å)	Avg. Number of Interactions (H-bond)
WT	29.30 ± .14	13 ± 1.40
K527A	29.77 ± .18	10 ± 1.05
Y736A	29.90 ± .17	09 ± 1.17
K527A_Y736A	30.30 ± .21	09 ± 1.05
D796A	29.01 ± .13	17 ± 2.11

Although mutant K527A is unable to hydrogen bond with either N794 or D796 as a result of the point mutation, the Y736-E730 contact close to the short loop linking the two domains persists throughout the simulation. Mutant Y736A does not form the hydrogen bond close to the short loop linking the two domains, and the interaction between the K527 amino group and the carboxylate O*δ* atom of D796 is also lost completely during equilibration. Only a contact between the K527 amino group and the backbone carbonyl O atom of N794 is persistent. Finally, analysis of H-bond numbers at the interface between helicase domains 1 and 2 during the simulations suggests a correlation with helicase cleft opening (Table 2). Thus, double mutant K527A_Y736A with the largest interdomain distance displays only up to nine interdomain H-bonds compared to ∼13 in WT HsdR, whereas closure of the cleft in mutant D796A is correlated with up to 18 H-bonds at the interdomain interface.

## Discussion

The structurally characterized homologs of HsdR are the RecA-like SF2 helicases for its translocation activity and the Type II restriction endonucleases for its endonuclease activity. Both these homologs carry out their enzyme activity as isolated proteins with no additional functional domains nor assembly with other subunits required. The HsdR subunit in contrast is a single polypeptide chain that represents a symbiotic fusion of two RecA-like helicase domains with folds characteristic of SF2 helicases, an endonuclease domain characteristic of the Type II enzymes, and an auxiliary C-terminal helical domain. Based on the results presented here, the role of the helical domain can now be said to include participation in all the individual activities of the HsdR motor subunit: DNA cleavage, DNA translocation, and ATP hydrolysis.

Because the observed interdomain interactions contribute to enzymatic function as described in this work, they may be conserved in other Type1 restriction-modification systems in which single HsdR subunits have multiple functions. Sequence alignment suggests that the main interaction partner identified here in helicase domain 2, K527, is conserved throughout Type I R-M families IA, IB, IC as part of a proposed new (Q/D)KT motif (Fig. 1C). A similar three-dimensional arrangement and function can be suggested for this region of these other Type I R-M systems. Other related translocases from the SF2 superfamily such as Rad54 display low sequence identity in this region, suggesting that the (Q/D)KT motif may be conserved specifically in the Type I R-M systems, which require interdomain contact between helicase domain 2 and the helical domain to integrate the functions of the domains.

The present work demonstrates a previously unsuspected role for the helical C-terminal domain of the HsdR motor subunit in DNA translocation, cleavage, and ATPase activities. This domain has been assumed to be involved in assembly with the HsdS/M methylase (Dryden et al., 2001; Obarska-Kosinska et al., 2008). A role involving all three of the subunit’s activities implicates the C-terminal domain in controlling enzymatic function. Thus a controlling function resides at a nexus of intersubunit communication.

##  Supplemental Information

10.7717/peerj.2887/supp-1 Supplemental Information 1 Structural models of WT and mutants in PDB format The file contains the pdb files of all created models of EcoR124I HsdR, WT as well as of each mutant. All initial models used for molecular dynamics and the final equilibrated structure after molecular dynamics are included for each structure.

## Additional Information and Declarations

### Data Availability

The following information was supplied regarding data availability: The raw data of experimental work is included in the manuscript as gels in Figs. 2A–2D, as data points in Figs. 3 and 4, and as the efficiency of plating in Table 2. The computational models of WT and the mutants have been supplied as a Supplemental File.
